# Prediction of promiscuous multiepitope-based peptide vaccine against RdRp of rotavirus using immunoinformatics studies

**DOI:** 10.1590/S1678-9946202466055

**Published:** 2024-09-06

**Authors:** Hailah M. Almohaimeed, Ahmed M. Abdulfattah, Fayez Alsulaimani, Aisha Alshammary, Mohammad Osama Almohaini, Khowlah Abdulrahman Almehiny, Almonther Abdullah Hershan, Abdullah Saleh Alkhamiss, Ruqaih S. Alghsham, Hanaa Ghabban, Mona H. Soliman, Jamal A. Alorabi, Waleed Al Abdulmonem

**Affiliations:** 1 Princess Nourah bint Abdulrahman University, College of Medicine, Department of Basic Science, Riyadh, Saudi Arabia; 2 King Abdulaziz University, Faculty of Applied Medical Sciences, Department of Medical Laboratory Sciences, Jeddah, Saudi Arabia; 3 Alyamamah Hospital, Pediatric Infectious Department, Riyadh, Saudi Arabia; 4 Riyadh Third Cluster, Family Medicine Department, Riyadh, Saudi Arabia; 5 Alyamamah Hospital-Riyadh, Second Health Cluster, Registrars Preventive Medicine and Public Health, Infection Control Department, Riyadh, Saudi Arabia; 6 University of Jeddah, College of Medicine, Department of Medical Microbiology and Parasitology, Jeddah, Saudi Arabia; 7 Qassim University, College of Medicine, Department of Pathology, Buraidah, Saudi Arabia; 8 Qassim University, College of Medicine, Department of Medical Microbiology and Immunology, Qassim, Saudi Arabia; 9 University of Tabuk, Faculty of Science, Department of Biology, Tabuk, Saudi Arabia; 10 Cairo University, Faculty of Science, Botany and Microbiology Department, Giza, Egypt; 11 Taibah University, Faculty of Science, Biology Department, Al-Sharm, Yanbu El-Bahr, Kingdom of Saudi; 12 Taif University, College of Science, Department of Biology, Taif, Saudi Arabia

**Keywords:** Promiscuous epitope prediction, MEV Rotavirus, RdRp of Rotavirus, *In silico* Rotavirus vaccine development, B- cell and T- cell epitopes prediction

## Abstract

Rotavirus, a dsRNA virus in the Reoviridae family, shows a segmented genome. The VP1 gene encodes the RNA-dependent RNA polymerase (RdRp). This study aims to develop a multiepitope-based vaccine targeting RdRp using immunoinformatic approaches. In this study, 100 available nucleotide sequences of VP1-Rotavirus belonging to different strains across the world were retrieved from NCBI database. The selected sequences were aligned, and a global consensus sequence was developed by using CLC work bench. The study involved immunoinformatic approaches and molecular docking studies to reveal the promiscuous epitopes that can be eventually used as active vaccine candidates for Rotavirus. In total, 27 highly immunogenic, antigenic, and non-allergenic T-cell and B-cell epitopes were predicted for the Multiepitope vaccine (MEV) against rotavirus. It was also observed that MEV can prove to be effective worldwide due to its high population coverage, demonstrating the consistency of this vaccine. Moreover, there is a high docking interaction and immunological response with a binding score of −50.2 kcal/mol, suggesting the vaccine’s efficacy. Toll-like receptors (TLRs) also suggest that the vaccine is physiologically and immunologically effective. Collectively, our data point to an effective MEV against rotavirus that can effectively reduce viral infections and improve the health status worldwide.

## INTRODUCTION

Rotavirus belongs to *Reoviridae* family and cause gastroenteritis. In the small intestine, villi is the site of virus replication in the cytoplasm of established enterocytes^1^. Rotavirus is more common in young children and newborns and can lead to severe dehydration and hospitalization. If untreated, rotavirus infection can be fatal^2^. Rotavirus is a segmented virus that shows a genome size of about 18 kb and 11 segments are surrounded by three shells. VP7 (glycoprotein or G protein) and VP4 protein, which is a separate structural protein, is not produced from polyprotein cleavage, are involved in the formation of the outer protective layer of rotavirus^3^.

Rotavirus is classified into nine species ranging from rotavirus A–I. Rotavirus A is the predominant species responsible for over 90% of rotavirus infections in children. It predominantly infects humans and stands as the leading cause of severe gastroenteritis among children and infants globally^4^.

Almost 111 million gastroenteritis patients are due to rotavirus infection and require home care. Moreover, clinical visits include 25 million, with 2 million hospitalizations each year. In developing countries, death rate among children is 80% each year due to rotavirus infection^5^. From October 1985 to April 1986, a study was conducted in local hospitals in Pakistan comparing various diagnosis methods for the identification of rotavirus in children’s feces with severe diarrhea. They found better results for enzyme-linked immunosorbent assay (ELISA), with 72.4% positive stool samples^6^. Rotavirus constitutes a significant cause of severe diarrhea among Saudi children, exhibiting a variable prevalence of infection ranging from 10% to 65.5%^7-9^. In our locality, Jeddah, previous investigations have demonstrated rotavirus prevalence rates ranging from 42% to 46% among hospitalized children with gastroenteritis, with peak levels observed during cooler months^7,9,10^. In a more recent study, rotavirus infection continued to emerge as the primary causative agent of gastroenteritis, accounting for 42.9% of all pediatric cases admitted with gastroenteritis to one of the largest tertiary hospitals in Jeddah^11^. A study conducted in Riyadh, Saudi Arabia, found rotavirus in 65.5% of diarrheal stool samples, showing a higher incidence among children aged 1 year or less, accounting for 81% (534 out of 660 cases), compared to those over 1 year of age, which represented 19% (126 out of 660 cases)^11^.

VP1 functions as an RNA polymerase enzyme localized within the core of the virus particle^12^. It catalyzes the synthesis of mRNA transcripts necessary for the production of viral proteins within the host cell and generates duplicates of the rotavirus genome RNA segments required for the assembly of newly formed virus particles^13,14^.

VP1 is a closely packed, globular protein, with a diameter of about 70 Å. It consists of three domains: the N-terminal domain (amino acids [aa] 1–332), the polymerase domain, which includes the fingers, thumb, and palm subdomains (aa 333–778)^15^, and the canonical motifs A–F^15,16^. There are four tunnels that extend to the catalytic part of the polymerase. One tunnel functions as the template entry tunnel, another as the nucleotide exchange tunnel, and the remaining two as RNA exit tunnels^17^. One of these RNA exit tunnels is considered active during replication; this tunnel extends to the bracelet domain and elucidates the pathway for the release of novel dsRNA. Epitope-based vaccines are a new approach used for inducing pathogen-specific immunity. Epitopes are identified and selected, acting as vaccine targets^18^. The main objective of this study is to use epitope prediction to assist in designing a multiepitope vaccine by mimicking the structure and function of natural epitopes^19,20^.

## MATERIALS AND METHODS

### Multiepitope vaccine construction (MEV)

Suitable linkers were combined with B-cell, T-cell epitopes, and an adjuvant, with the latter being employed to enhance the vaccine immunogenicity. When peptides are chosen individually, they may exhibit low immunogenicity. The EAAAK linker was employed to merge the initial CTL epitope with the adjuvant, and also to segregate the domains of a bi-functional fusion protein. GPGPG and AAY-linkers were utilized to combine HTL and CTL-epitopes and to facilitate identification of epitopes.

### Structural analysis of MEV

The homology of the vaccine against human proteome was checked via BLASTp. Physiochemical properties were assessed using the ProtParam server, including molecular weight (MW), theoretical isoelectric point (pI), instability index (II), aliphatic index (AI), Grand Average of Hydropathicity (GRAVY), and half-life. It is a tool that estimates various physical and chemical properties for a protein saved in Swiss-Prot or TrEMBL, or for a protein sequence provided by user. IEDB immunogenicity tool and Vaxijen 2.0 server were used to assess immunogenic and antigenic profiles. The AllerTOP server was used to analyze vaccine’s allergenic reaction. Secondary structure of the vaccine was determined via PSIPRED workbench and SOPMA. The Self Optimized Prediction method with alignment (SoPMA) is an online tool used to predict secondary structures of a protein.

### Three-dimensional protein structure prediction and refinement of tertiary structure

A protein’s 3D structure represents its lowest energy structure, in which it undergoes precise twisting and bending to achieve maximum stability. This vaccine’s structure was modeled using the server, which leverages connectivity information to improve the structural accuracy of proteins. I-TASSER was also used for structure based annotation and protein structure prediction. The refinement and optimization of the 3D structure of the vaccine were conducted using the Galaxy Refine server. The refined structure was validated using the RAMPAGE server, which generates the Ramachandran plot. Moreover, the ProSA-web was employed to assess overall quality score. The ERRAT server was used for data review on non-bonded interactions.

### MEV docking with immune receptor and MHC molecules

After the interaction with the host’s immune cells, the vaccine elicits an effective immune response. Molecular docking was performed to observe the binding capacity of the vaccine with human immune receptors and the MHC molecule TLR3. The structure of TLR3 (PDB ID: 3CIG) was retrieved from Protein Data Bank. For docking, the HADDOCK2.4 server was used, which is based on Ambiguous Interaction Restraints (AIRs). For the visualization of docked complexes, PyMOL1.3 server was utilized.

### Molecular dynamic simulation

*In silico* research, molecular dynamics play a crucial role in assessing the integrity of protein-protein complexes. The fundamental protein dynamics can be contrasted with standard nodes to ascertain the stability of the protein. In internal coordinates, collective protein motion was explained by using IMODS server. The collective functional movements of biological macromolecules are naturally reproduced by normal mode analysis (NMA) in internal coordinates. Even with huge macromolecules, IMODS allows the exploration of such modes and produces feasible transition routes between two homologous structures. The server quantified the internal motions of the complexes by describing their covariance, deformability, B-factors, and eigenvalues. Stiffness of motion indicated the value of standard mode. When the eigenvalue is low, it aids in the deformation of structures with particular energy.

### Immune simulation

An *in silico* immunological simulation was performed using the C-ImmSim-10.1 server to validate the MEV immunological response. In presence of antigens in mammalian immune system, both humoral and cellular response are determined at cellular scale via c-Immsim model. It is an online approach which is easily available for researchers. The capacity of the MEV to simulate immune cells such as CTL, HTL, dendritic cells, B-cells, cytokines, NK cells, and immunoglobulins was examined. In clinical practice, a four-week gap between two vaccine doses is suggested. Hence, two doses were administered with a four-week interval between them, and the simulation was run for 1,000 steps.

### In silico cloning and optimization of codons

The MEV sequence was reverse translated using the JCat server to obtain a cDNA sequence of the gene, which was then subjected to codon optimization. For the most sequenced prokaryotic and selected eukaryotic organisms, a simple method to adapt codon usage is presented by the Codon Adaptation Tool. Codon optimization considered the GC concentration of the cDNA sequence, as well as the Codon Adaptation Index (CAI). CAI values should be in the range of 0 to 1, with greater CAI values implying better gene expression. The GC content should range from 30% to 70%. The adapted sequence was finally cloned to pET-30(+).

**Figure 1:**
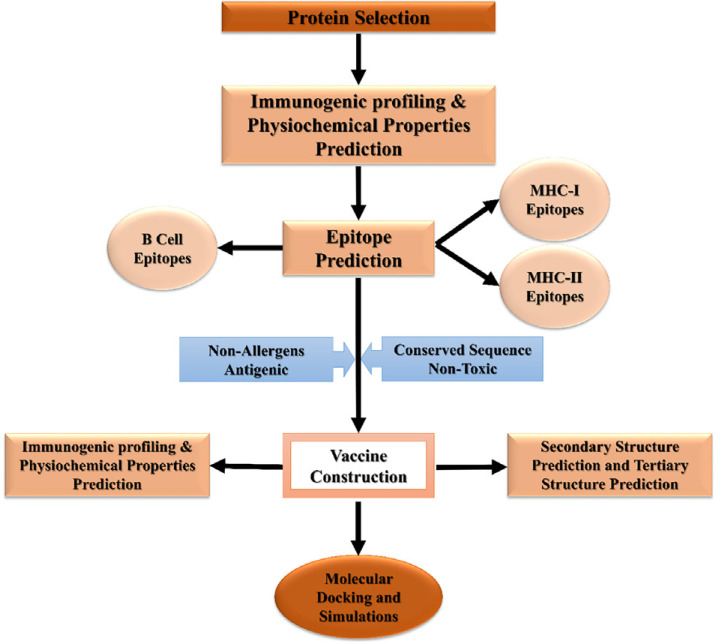
Schematic representation of the prediction of MEV against rotavirus.

Figure 1 shows the schematic representation of the strategies adopted to predict promiscuous MEV against rotavirus.

## RESULTS

### Epitopes evaluation and selection

A total of 39 CTL epitopes were generated from the target protein of rotavirus. The top 11 high antigenic, immunogenic, non-toxic, and non-allergenic epitopes were chosen to develop the MEV. In total, 27 specific HTL epitopes were generated. The top seven epitopes were chosen for vaccine by analyzing their cytokines capability (IL-10, IL-4, and IFN-Gamma). Similarly, 15 B-cell epitopes were also selected, from which nine epitopes were finalized for synthesis of vaccine by evaluating their allergenicity, toxicity, and immunogenicity, as shown in Table 1.

**Table 1:** Final CTL , HTL, and B-Cell selected epitopes for the construction of a vaccine against rotavirus.

CTL Epitopes				
Epitope	Alleles	Position	Antigenicity	Immunogenicity
TTENLELEY	HLA-A*31:01	260	0.9404	0.02297
LLSVNYGMY	HLA-A*23:01 HLA-C*07:02 HLA-C*14:02 HLA-A*24:02	989	1.9021	0.36777
YSLSFHVGY	HLA-B*57:01 HLA-B*58:01 HLA-A*01:01	232	1.8656	0.02653
VYDFEDVTY	HLA-C*07:02 HLA-B*39:01 HLA-B*48:01	340	1.3380	0.41831
STDVKNATR	HLA-B*44:02 HLA-B*44:03 HLA-B*57:01	154	1.3921	0.23133
YSSKSVNYL	HLA-A*02:01 HLA-A*32:01	268	0.5371	0.51613
LVDLNSAKF	HLA-C*14:02 HLA-A*24:02	999	0.4371	0.02357
SFAELMLKY	HLA-B*44:02 HLA-B*44:03	471	1.2463	0.01245
SVHASKTDY	HLA-A*31:01	1080	0.5798	2.3587
ASRYLKFVY	HLA-B*44:02 HLA-B*44:03	6	1.2357	2.3546
TFPNIALIY	HLA-C*14:02 HLA-A*24:02	315	0.8935	2.0243
**HTL Epitopes**				
**Epitope**	**Alleles**	**Position**	**Antigenicity**	**IFN-Y**
AVFMIDLALRLKVIN	HLA-DRB1*03:09 HLA-DRB1*03:05 HLA-DRB1*03:01 HLA-DRB1*03:06 HLA-DRB1*03:07 HLA-DRB1*03:08 HLA-DRB1*11:28 HLA-DRB1*13:05 HLA-DRB1*11:07	1018-1032	0.9915	Positive
KTPTAVFMIDLALRL	HLA-DRB1*09:01 HLA-DRB1*07:03 HLA-DRB1*11:02 HLA-DRB1*11:21 HLA-DRB1*13:22	318-332	1.1174	Positive
NIALIYSLSFHVGYR	HLA-DQA1*01:01/DQB1*05:01	1020-1034	0.6977	positive
PTAVFMIDLALRLKV	DRB1*03:06 HLA-DRB1*03:07 HLA-DRB1*03:08	1042-1056	0.2547	Positive
TAVFMIDLALRLKVI	DRB1*03:05 HLA-DRB1*03:01 HLA-DRB1*03:06	1019-1033	0.3578	Positive
TPTAVFMIDLALRLK	HLA-DQA1*01:01/DQB1*05:01	319-333	1.2347	Positive
IALIYSLSFHVGYRK	DRB1*03:01 HLA-DRB1*03:06	378-692	1.3659	Positive
**B-Cell Epitopes**				
**Epitope**	**Position**	**Score**	**Antigenicity**	**Immunogenicity**
HKRYTTNIPPVDERNP	427	0.58	1.0434	0.06495
MFTISEMKSTDVKNAT	146	0.52	0.9433	0.1772
YDFEDVTYQNNYFVTD	14	0.85	1.7077	0.49894
KRNQDSSYDMAATLYA	692	0.68	1.7424	0.19838
HVGYRKQALSDAVYDQ	328	0.68	1.1223	0.05981
YFGLRTHDYDIKGSSS	906	0.57	1.2357	0.35678
NSSIALPKEENNTMPL	880	0.36	1.3579	0.3675
MEMYKEYSERIENEIF	354	0.24	1.0254	0.4753
TVTYDDNVNMEMYKEY	345	0.92	1.3547	0.3587

### Immunogenic and physiochemical profiling and population coverage analysis

**Figure 2:**
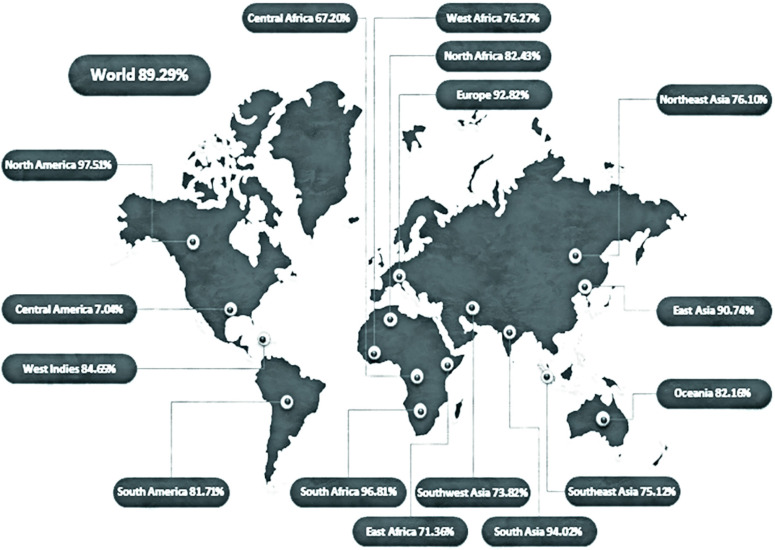
Worldwide population exposure of MEV epitopes.

The immunogenic and physiochemical properties of the synthesized vaccine are studied further. Upon comparing the homology of the produced vaccine to the human proteome, the findings showed no similarity with any human proteome region. After that, vaccine’s allergenicity, antigenicity, and toxicity was measured, showing that it is extremely antigenic, non-toxic, and non-allergenic. ProtParam was used to test physiochemical properties. The vaccine’s molecular weight was 40,736.58 kDa, whereas the theoretical PI was 9.16 kDa. Moreover, it shows a mean half-life of 30 h *in vitro*, being > 20 h *in vivo* (Yeast), and > 10 h *in vivo* (*E. coli*). The GRAVY (grand average hydropathicity) was estimated to be −0.331. Population coverage is an essential parameter for vaccine construction, as different ethnic groups and geographical circles in the world share different distributions of HLA alleles. This study determined the total population coverage of selected T cell epitopes with related HLA alleles. For the selected epitopes, combined coverage was measured as ~89% of the world’s population. The highest population coverage (92.82%) was observed in Europe (Figure 2).

### MEV 3D structure prediction, evaluation, and molecular docking analysis

**Figure 3:**
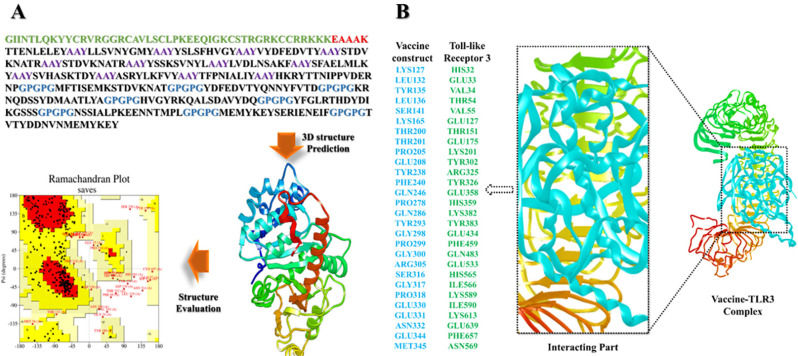
A) Primary sequence of the MEV; 3D model of the MEV; Ramachandran plot of the MEV construct; B) Intermolecular binding mode and interacting residues.

The I-TASSER was used to predict the tertiary structure of the vaccine, in which c-score was estimated as −1.56. The predicted structure underwent refinement in the Galaxy Refine server. The Ramachandran plot analysis of the refined model revealed that 60.5% of amino acids were situated within favored regions, 31.3% in the allowed regions, and 3.1% in the outlier regions. Z score was estimated as −1.01. The optimized model achieved a score of 92.8968 in ERRAT quality check analysis. These results indicate that the refined model exhibited excellent quality. (Figure 3A). An effective interaction between the immune receptor and the antigen molecule is essential for active and immune response. The HADDOCK version 2.4 was employed for the docking between the vaccine and TLR3. By following the bacterial recognition, TLR4 can produce efficient immune response. The docking results indicate the strong interaction between vaccine and TLR3. The binding score between TLR3 and the vaccine was estimated to be −50.2 kcal/mol (Figure 3B). A total of 10 hydrogen bonds were found between TLR3 and MEV within a range of 3.34 Å.

### Molecular dynamics simulation and immune simulation

**Figure 4:**
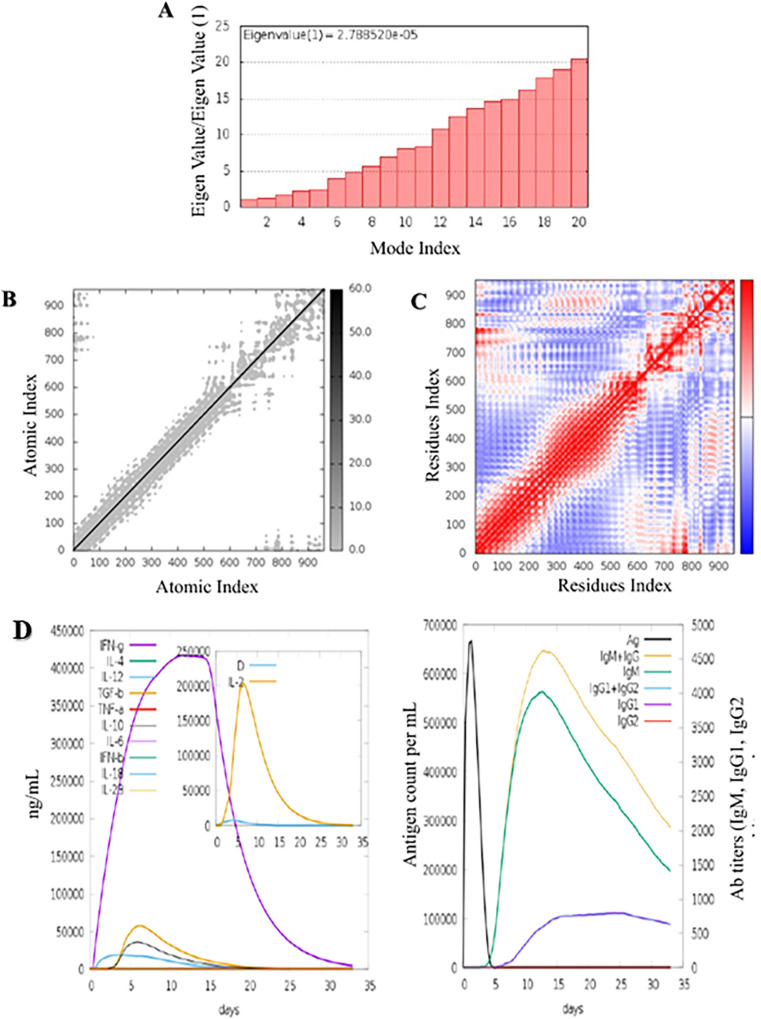
Molecular simulation dynamics analysis of the MEV with TLR3: A) Eigenvalue assessment of the complex trajectory of the MEV construct with TLR3; B) Atomic fluctuation analysis of the MEV-TLR3 complex using B-factors; C) Covariance matrix analysis of the complex system; D) Immunoglobulins response per mL to the presence of MEV antigen; Interleukins and interferon concentration in ng/mL generated in response to MEV.

Normal Mode Analysis (NMA) was used to examine protein mobility and stabilization on a large scale. Molecular dynamics simulations were conducted to assess the stability and interactions of the vaccine with TLR3. Using the IMODS server, we evaluated protein mobility and stability, highlighting significant eigenvalue and B-factor correlations. The B-factor value was directly proportional to RMS due to the normal mode analysis (Figure 4A). The elastic map illustrated atoms connected by springs, in which each point represented a spring, and regions of higher stiffness were depicted in grey color, with intensity corresponding to the level of stiffness (Figure 4C). A covariance matrix portrays the relationships between residue pairs, with different pairs of correlated, disassociated, or irrelevant motions represented in different colors, including reddish, bluish, and white (Figure 4B). The immunogenic profile of the designed MEV was evaluated using the C-IMMSIM server. All primary, secondary, and tertiary immune responses showed significant contributions to vaccine immunity. High titers of IgG + IgM antibodies were found, followed by IgM and IgG1. Different B cell isotypes were developed in response to vaccine administration, resulting in the development of memory cells. Moreover, the vaccine candidate stimulates high levels of IFN-γ and IL-2 (Figure 4D).

### In silico cloning of MEV in Escherichia coli pET-30a vector

**Figure 5:**
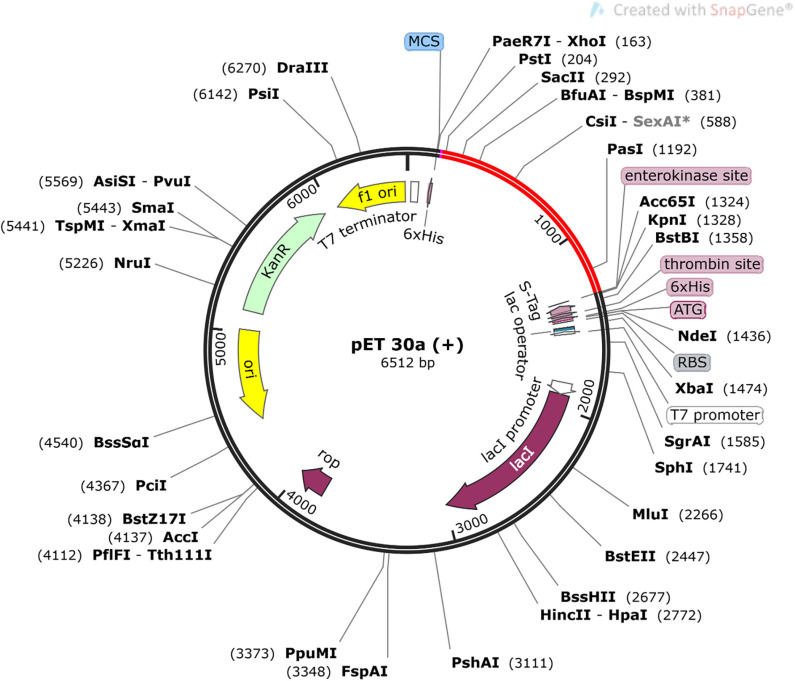
Cloned sequence of MEBSV (colored as red) into pET-30a (+) expression vector.

Codon optimization and *in silico* cloning were used to ensure that MEV protein was efficiently expressed in the *E. coli* host system. CAI score was 0.95 and GC content was 52.97% in improved cDNA sequence. The synthesized codon was inserted between the Ncol and Xhol restriction sites in the *E. coli* vector pET-30a (+), as shown in Figure 5. The size of the clone was 6,512 bp.

## DISCUSSION

Rotavirus is a deadly viral infection that targets infants, especially in underdeveloped countries^21^. Rotarix and RotaTeq are the rotaviral vaccines used to reduce rotavirus infection. To reduce the disease burden, live attenuated vaccines with human strains have been designed, which led to successful trials showing positive results. These Rotavirus vaccines are expensive and difficult to handle. Moreover, it has been observed that countries that have adopted rotavirus vaccination in their national vaccination program show significantly reduced frequency of morbidity and mortality due to rotavirus infection.

Recently, peptide based vaccination is of great importance for researchers^22^. Although, ligand-based antivirals also pave a way towards better health quality, peptide based vaccines are given preference due to multiple factors such as reduced side effects, less toxicity, possibility of visualizing cellular dynamics, and rapid action. In this regard, immunoinformatics tools are of remarkable importance because they can pinpoint the cellular check points without the aid of expensive and time consuming wet lab based tool^23,24^. These tools enable the researchers to have a dynamic and rapid solution to the complicated biological systems^25,26^.

In this study, we predicted MEV for rotavirus infection. The results suggested 11 promiscuous CTL epitopes because of their high antigenicity and non-toxic nature. Moreover, the study predicted nine B-cell epitopes that can be considered as highly effective vaccine candidates due to their allergenicity, toxicity, and immunogenicity. To further strengthen the study, population coverage analysis was performed, revealing that the predicted MEV holds remarkable world coverage of about 89%, indicating that it can be used worldwide.

The predicted MEV construct reveals high binding affinity and immunogenic interactions. Similarly, previous studies have been conducted using various *in silico* tools to design effective vaccine candidates against viral infections targets^27-29^.

This study supports epitope-based vaccine design due to its effectiveness in targeting conserved regions. In contrast, the currently used monovalent live attenuated vaccine for rotaviruses contains only a single strain^30^. As a result, the variation in strains across different regions can reduce the vaccine’s efficacy.

## CONCLUSION

Recent studies have shown that multi-subunit vaccines are highly effective. Thus, this study suggests using immunoinformatics-based multiepitope prediction to develop a vaccine that displays a high level of antigenicity and can potentially be used for lab-based assessment of its therapeutic efficacy against rotavirus infection. This study highlights the potential of an immunoinformatics-based multiepitope vaccine with high antigenicity against rotavirus. The predicted vaccine can be further assessed for its therapeutic efficacy in laboratory settings.
